# Racial and socioeconomic disparities in postoperative outcomes following coronary artery bypass grafting: a national inpatient analysis

**DOI:** 10.1186/s43044-025-00675-7

**Published:** 2025-08-12

**Authors:** Abdul Hadi Khan, Bushra Ubaid, Elias Abboud, Anggie Lorena Renteria Chamorro, Daniela Alejandra Renteria Chamorro, Kevin Camilo Mejia Rios, Rodrigo Sandoval Martínez, Peter Collins, Raheel Ahmed

**Affiliations:** 1 Jinnah Sindh Medical University, Karachi, Pakistan; 2 Dow University of Health Sciences, Karachi, Pakistan; 3 Saint Joseph University of Beirut, Beirut, Lebanon; 4 Universidad Santiago de Cali, Palmira, Colombia; 5 Instituto Tecnológico y de Estudios Superiores de Monterrey, Monterrey, Mexico; 6 National Heart and Lung Institute Imperial College London, London, UK

**Keywords:** Ischemic heart disease, Coronary artery bypass grafting, Racial disparities, Socioeconomic status, In-hospital outcomes, Health equity

## Abstract

**Background:**

Ischemic heart disease (IHD) is the leading cause of death in adults and poses a substantial economic burden in the United States. Coronary artery bypass grafting (CABG) remains the standard surgical intervention for multivessel and left-main coronary disease. However, the combined impact of race and socioeconomic status on CABG outcomes has not been fully explored.

**Methods:**

A total of 47,373 admissions of adults (18–85 y) who underwent CABG from 2016–2020 were analysed. Adults aged 18–85 years with a primary diagnosis of IHD who underwent CABG were identified using ICD-10 codes. Data on patient demographics, socioeconomic indicators (household income quartile, insurance type), comorbidities (Charlson Comorbidity Index), and hospital characteristics were collected. Multivariable logistic regression models adjusted for clinical and hospital factors were used to estimate adjusted odds ratios (aORs) for in-hospital mortality, nonhome discharge, prolonged length of stay (> 75th percentile), and postoperative complications. Linear regression assessed differences in hospital costs.

**Results:**

Compared to White patients, Black individuals had significantly higher odds of nonhome discharge (aOR 1.37), prolonged hospitalization (aOR 1.54), and postoperative complications (aOR 1.35) (all *p* < 0.001). Hispanic and Asian/Pacific Islander patients also faced increased risks of prolonged stay (aORs 1.23–1.26) and complications (aORs 1.15–1.19) (all *p* < 0.001). Minority groups incurred significantly higher hospitalization costs, with adjusted increases ranging from $17,000 to $73,000 per admission (*p* < 0.001). Trends toward elevated in-hospital mortality in Native American and Black patients did not reach statistical significance.

**Conclusions:**

Racial and socioeconomic disparities persist in CABG outcomes and hospital resource utilization, despite adjustments for clinical and institutional factors. These findings underscore the need for targeted strategies to improve equity in cardiovascular surgical care, including enhanced access to preventive services, perioperative support, and system-level quality improvements.

**Highlights:**

- Non-Hispanic Black and Hispanic patients experience higher postoperative complication rates and longer hospital stays after CABG.
- Native American and Black patients showed trends toward higher in-hospital mortality, though not statistically significant.
- Patients from socioeconomically disadvantaged backgrounds incur significantly higher hospital costs and are more likely to experience prolonged hospitalizations.
- Racial and socioeconomic disparities persist despite adjustment for comorbidities and hospital-level factors.

**Supplementary Information:**

The online version contains supplementary material available at 10.1186/s43044-025-00675-7.

## Background

Ischemic heart disease (IHD) remains the leading cause of death in the United States, accounting for nearly 30% of all deaths among individuals over 35 years old [1]. Despite advances in prevention and treatment, the burden of IHD persists, both in terms of clinical outcomes and economic costs [2, 3].

Among revascularization strategies, coronary artery bypass grafting (CABG) is the treatment of choice for patients with complex CAD, including left main or triple-vessel involvement. Since its introduction, CABG has evolved significantly, with contemporary techniques yielding low in-hospital mortality and favorable long-term survival [4, 5]. However, despite these advancements, stark disparities in CABG outcomes persist.

Black patients continue to experience higher postoperative mortality and complication rates compared to White patients, even after accounting for comorbidities and hospital-related factors [6, 7]. Socioeconomic disadvantage further compounds these disparities, with patients from low-income or underserved communities facing worse outcomes, including longer hospital stays and increased rates of stroke and renal failure [8, 9]. Notably, hospitals serving predominantly non-White populations often have lower procedural volumes and quality metrics, contributing to nearly one-third of the observed mortality differences [10].

While previous studies have examined racial and socioeconomic disparities independently, there is a paucity of research exploring their combined effect on CABG outcomes. Understanding these intersecting factors is critical for informing equitable healthcare delivery.

This study aims to fill this gap by conducting a national inpatient analysis of racial and socioeconomic disparities in postoperative outcomes among IHD patients undergoing CABG. The findings aim to guide clinical strategies, inform policy, and support targeted interventions to reduce disparities in surgical care.

## Method

### Study design and data source

This retrospective observational study leverages the US Nationwide Inpatient Sample (NIS), the largest publicly available all-payer inpatient database, which systematically records hospitalization data. The NIS aggregates state-level inpatient information, capturing approximately 8 million admissions annually from nearly 1,050 hospitals across 44 states. Structured as a stratified 20% sample of US community hospitals according to the American Hospital Association, the database yields weighted estimates representing over 35 million hospitalizations nationwide. It includes detailed patient-level data, such as primary and secondary diagnoses, procedural codes, admission and discharge statuses, demographic profiles, expected payer information, length of stay (LOS), and hospital characteristics, thereby enabling robust national analyses of inpatient care [20]. Given that this research involves a secondary analysis of de-identified NIS data without direct patient or public engagement, it qualifies for exemption from institutional review board approval.

### Study population

Of 50,215 CABG admissions, 2,842 (5.6%) were excluded for missing age, gender, or in-hospital outcome data, yielding a final analytic cohort of 47,373 admissions. This retrospective cohort study included adult patients (18–85 years) with a primary diagnosis of IHD who underwent CABG as identified using ICD-10 procedure codes *(see supplementary Table 1).* Data were drawn from a nationally representative inpatient database spanning the years 2016 to 2020. Individuals with missing key data elements such as age, gender, or primary outcomes were excluded from the analysis. For variables with small proportions of missingness (e.g., insurance status, household income), we retained the cases in the dataset, and missing values were handled using appropriate imputation or included as separate"missing"categories in descriptive analyses where applicable.

### Study variable and outcome measure

The study utilized the NIS to obtain detailed data at both the patient and hospital levels. Patient-related independent variables included demographics such as age (categorized as 18–44, 45–64, and ≥ 65 years) and race/ethnicity (classified as White, Black, Hispanic, Asian or Pacific Islander, Native American, and Other) as well as socioeconomic indicators like insurance status and household income quartile. The ‘Other’ race/ethnicity category comprises individuals coded as multiracial, Other specified,’ or ‘Unknown’ in the NIS race field, representing ≈ 2.8% of the cohort. Additionally, clinical characteristics were examined, with comorbidity burden quantified using the Charlson Comorbidity Index (CCI) (stratified into 0, 1, 2, and ≥ 3). Hospital-level factors, including location/teaching status and bed size, were also incorporated into the analysis.

The primary endpoint was in-hospital mortality among IHD patients undergoing CABG. Secondary endpoints included discharge to a non-home setting (i.e., discharge to a skilled nursing facility, rehabilitation center, or hospice), extended LOS, in-hospital complications, and overall hospital costs. Prolonged LOS was de-fined as > 11 days, corresponding to the 75th percentile of the study cohort’s LOS distribution. In-hospital complications were identified using predetermined ICD-10-CM diagnostic and procedural codes, as detailed in *Supplementary Table 2*. Hospital costs were evaluated as a continuous variable. Because the frequency of specific complications was low in certain racial strata, major post-operative complications were analyzed as a composite endpoint to maximize statistical power and provide a stable measure of overall peri-operative morbidity. Because the five-year study window (2016–2020) entails < 10% cumulative medical-care inflation and affects all groups uniformly, costs were analysed in nominal dollars consistent with prior NIS cost studies.

## Statistical analysis

All statistical analyses were performed using R version 4.4.2. Descriptive statistics were calculated for patient demographics, clinical characteristics, and hospital factors. Continuous variables were summarized as means ± standard deviations or medians with interquartile ranges (IQR), as appropriate based on their distribution, and categorical variables were presented as frequencies and percentages. Group comparisons for categorical variables were performed using chi-square tests, while continuous variables were compared using the Kruskal–Wallis test when the normality assumption was not met and analysis of variance (ANOVA) when data were normally distributed.

Multivariable logistic regression models were used to assess the independent association between race/ethnicity and binary clinical outcomes including in-hospital mortality, unfavourable discharge, prolonged LOS, and complications with White patients serving as the reference group. Each model was adjusted for potential confounders, including age, insurance status, household income, hospital location and teaching status, and comorbidities. Adjusted odds ratios (aOR) with 95% confidence intervals (CI) and corresponding p-values were reported.

For the analysis of hospital costs, a multivariable linear regression model was employed to estimate the adjusted difference in mean hospital costs across racial/ethnic groups, controlling for the same set of covariates. A two-tailed p-value of < 0.05 was considered statistically significant.

## Result

Among 47,373 CABG admissions from 2016‒2020, 78.0% were White (n = 36,956), 7.0% Black (n = 3,316), 8.2% Hispanic (n = 3,878), 3.3% Asian/Pacific Islander (n = 1,580), 0.7% Native American (n = 302), and 2.8% were classified as other race/ethnicity (n = 1,341). Table 1 details the demographic, clinical, and hospital characteristics of IHD patients undergoing CABG, stratified by race. Statistically significant differences were observed across all variables (p < 0.001). Overall, the study population was predominantly elderly, with 55.3% of patients aged 65 years and older and only 2.0% in the 18–44 age group. When stratified by race, White patients dominated the older age category (64.8% were ≥ 65), whereas Black, Hispanic, and Native American patients exhibited a relatively higher proportion in younger age groups. Insurance status also varied significantly: while Medicare/Medicaid was the primary payer for 79.1% of the overall cohort, private insurance was more prevalent among White and Asian/Pacific Islander patients. Furthermore, household income distribution revealed socioeconomic disparities—patients from lower-income quartiles were more frequently represented among Black and Hispanic groups, whereas White and Asian/Pacific Islander patients were more likely to belong to higher-income quartiles. Differences in admission type were evident across races. Elective admissions were more common among White and Asian/Pacific Islander patients, while Black, Hispanic, and Native American patients experienced significantly higher rates of non-elective admissions, suggesting variations in disease presentation or access to planned care. Notably, the proportion of patients with a CCI ≥ 3 was highest among Black (25%) and Hispanic (23%) patients, compared with 14% in Whites. White patients were more frequently treated in large, urban teaching hospitals, in contrast to Black, Hispanic, and Native American patients, who were more likely to be admitted to smaller or urban nonteaching facilities. In addition, the distribution of weekend versus weekday admissions differed significantly by race.

**Table 1:** Demographic and Clinical Characteristics of Patients with Ischemic Heart Diseases undergoing Coronary Artery Bypass Grafting

Characteristics	Total	White	Black	Hispanic	Asian or Pacific Islander	Native American	Others**	p-value
**Demography**								
***Age, (y)***								** < 0.001**
18–44	994	641	120	127	46	9	51	
45–64	19,062	14,076	1,672	1,889	698	130	597	
65 +	27,317	22,239	1,524	1,862	836	163	693	
***Insurance Status***								** < 0.001**
Medicare/Medicaid	29,309	23,002	2,064	2,358	902	186	797	
Private Insurance	14,938	11,681	951	1,176	598	83	449	
Self-pay/No charge/Others	3,045	2,212	289	340	79	31	94	
Missing	81	61	12	4	1	2	1	
***Household Income***								** < 0.001**
Quartile 1	13,433	9,722	1,674	1458	159	124	296	
Quartile 2	12,751	10,374	719	988	281	75	314	
Quartile 3	11,322	9,135	526	825	435	45	356	
Quartile 4	9,046	7,118	330	524	682	40	352	
Missing	821	607	67	83	23	18	23	
***Admission Type***								** < 0.001**
Elective	24,631	18,187	1,353	1,597	729	137	537	
Non-Elective	22,540	18,618	1,951	2,257	847	164	794	
Missing	202	151	12	24	4	1	10	
CCI*								** < 0.001**
0	7,679	6,477	354	493	228	32	216	
1	11,776	9,363	664	922	407	64	356	
2	9,988	7,833	659	808	329	74	285	
3 +	17,809	13,283	1,639	1,655	616	132	484	
**Hospital Characteristics**								
***Hospital bed size***								** < 0.001**
Small	5,909	4,761	383	405	151	65	144	
Medium	12,870	10,008	899	1,166	354	77	366	
Large	28,594	22,187	2,034	2,307	1,075	160	831	
***Hospital location/teaching status***								** < 0.001**
Rural	1,465	1,324	98	13	5	18	7	
Urban nonteaching	7,850	6,221	393	691	285	60	200	
Urban teaching	38,058	29,441	2,825	3,174	1,290	224	1,134	
***Weekend Admission***								** < 0.001**
Yes	5,353	4,022	421	513	193	40	164	
No	42,020	32,934	2,895	3,365	1,387	262	1,177	

*Charlson Comorbidity Index

^**^ ‘Other’ includes multiracial, other-specified, and unknown race/ethnicity as per NIS coding

Statistically significant value having value 0.05 are highlighted in bold

## Clinical outcomes stratified by race and ethnicity

Table 2 summarizes the postoperative outcomes of patients with IHD undergoing CABG, stratified by race/ethnicity. Statistical comparisons revealed significant differences across multiple domains.

**Table 2:** Postoperative Outcomes of Ischemic Heart Disease Patients who underwent Coronary Artery Bypass Grafting Stratified by Race and Ethnicity

Outcomes	Patients, No. (%)						
	White 36,956 (78.0%)	Black 3,316 (7.0%)	Hispanic 3,878 (8.2%)	Asian or Pacific Islander 1,580 (3.3%)	Native American 302 (0.7%)	Others 1,341 (2.8%)	p-value
In-hospital Mortality	607 (1.6)	73 (2.2)	79 (2.0)	28 (1.7)	10 (3.3)	25 (1.9)	0.026
Unfavorable Discharge*	21,510 (58.2)	2,094 (63.1)	2,096 (54.0)	954 (60.4)	122 (40.1)	744 (55.5)	< 0.001
Prolonged LOS *†	7,424 (20.1)	1,036 (31.2)	978 (25.2)	367 (23.2)	35 (21.5)	331 (24.7)	< 0.001
Complications	11,919 (32.2)	1,377 (41.5)	1,439 (37.1)	569 (36.0)	106 (35.0)	478 (35.6)	< 0.001
Hospital Cost (Median, IQR)	$159 K (114 K-230 K)	$176 K (125 K–259 K)	$227 K (158 K–336 K)	$211 K (144 K–324 K)	$163 K (115 K–237 K)	$203 K (136 K–303 K)	< 0.001

*Patients who died in the hospital (n = 822) were excluded

*†Length of stay (LOS) exceeds the 75th percentile, which is 11 days

### In-hospital mortality

Although the overall in-hospital mortality rate was low, significant racial disparities were observed. White patients had an in-hospital mortality rate of 1.6%, whereas Black patients experienced a higher rate of 2.2%. Hispanic and Asian/Pacific Islander patients both exhibited mortality rates of 2.0% and 1.7%, respectively. Notably, Native American patients had the highest mortality rate at 3.3%, while patients categorized as Others had a rate of 1.9%. Although crude mortality appeared higher in Native-American and Black patients, these differences did not reach statistical significance after multivariable adjustment (all p > 0.05) and should be interpreted as trends rather than definitive disparities.

### Unfavourable discharge

Among survivors, unfavourable discharge outcomes defined as transfers to skilled nursing facilities or rehabilitation centres differed markedly by race (p < 0.0001). Specifically, 11.0% of White patients were discharged unfavourably compared to 16.1% of Black patients, 14.0% of Hispanic patients, 13.4% of Asian/Pacific Islander patients, 12.1% of Native American patients, and 13.4% of patients classified as Others.

### Prolonged length of stay (LOS)

Prolonged LOS, defined as a stay exceeding the 75th percentile (> 11 days), also varied significantly by race (p < 0.0001). Prolonged LOS was observed in 20.1% of White patients; however, a substantially higher proportion of minority patients experienced prolonged stays, with rates of 31.2% in Blacks, 25.2% in Hispanics, 23.2% in Asians/Pacific Islanders, 21.5% in Native Americans, and 24.7% in Others.

### Complications and hospital costs

The incidence of postoperative complications differed significantly by race (p < 0.001), with complications occurring in 32.2% of White patients, 41.5% of Black patients, 37.1% of Hispanic patients, 36.0% of Asian/Pacific Islander patients, 35.0% of Native American patients, and 35.6% of patients in the Others category. In addition, significant disparities in hospital costs were evident (p < 0.0001). The median hospital cost per $1,000 was lowest among Whites at $159 K (IQR: $114 K–$230 K) and was highest among Hispanic patients, with intermediate costs observed in Black, Asian/Pacific Islander, Native American, and other patients.

## Subgroup analysis

Table 3 displays the aOR for key postoperative outcomes among IHD patients undergoing CABG, with all models adjusted for age, primary payer, household income, hospital location/teaching status, and comorbidity burden (CCI category). White patients served as the reference group.

**Table 3:** Adjusted Odds Ratios (aOR) for Clinical Outcomes in Ischemic Heart Diseases Patients undergoing Coronary Artery Bypass Grafting, Stratified by Race and Adjusted for Demographic and Clinical Factors

	Black ^a^		Hispanic ^a^		Asian or Pacific Islander ^a^		Native American ^a^		Others ^a^	
	aOR (95% CI)	p-value	aOR (95% CI)	p-value	aOR (95% CI)	p-value	aOR (95% CI)	p-value	aOR (95% CI)	p-value
In-hospital Mortality	1.25 (0.96–1.59)	0.082	1.23 (0.95–1.55)	0.093	1.20 (0.80–1.73)	0.344	1.86 (0.91–3.34)	0.056	1.23 (0.80–1.80)	0.312
Unfavorable Discharge	**1.37 (1.27–1.48)**	** < 0.001**	0.91 (0.85–0.97)	0.0006	1.07 (0.96–1.20)	0.204	**0.47 (0.36–0.60)**	** < 0.001**	0.92 (0.82–1.04)	0.180
Prolonged LOS	**1.54 (1.41–1.67)**	** < 0.001**	**1.23 (1.13–1.33)**	** < 0.001**	**1.26 (1.11–1.43)**	** < 0.001**	0.96 (0.72–1.29)	0.822	**1.32 (1.16–1.51)**	** < 0.001**
Complications	**1.35 (1.25–1.46)**	** < 0.001**	**1.19 (1.10–1.28)**	** < 0.001**	**1.15 (1.03–1.29)**	**0.012**	1.02 (0.78–1.31)	0.879	1.18 (1.05–1.33)	0.006
Hospital Cost ^b^	**$17,392.3**	** < 0.001**	**$73,447.4**	** < 0.001**	**$72,074.5**	** < 0.001**	$312.3	0.974	**$52,055.8**	** < 0.001**

^a^ Compared with White patients and adjusted for age, primary payer, household income, hospital location region, teaching status, and comorbidities

^b^ Linear regression is utilized to predict hospital costs. Statistically significant value having p-value <0.05 are highlighted in bold

**Fig. 1:**
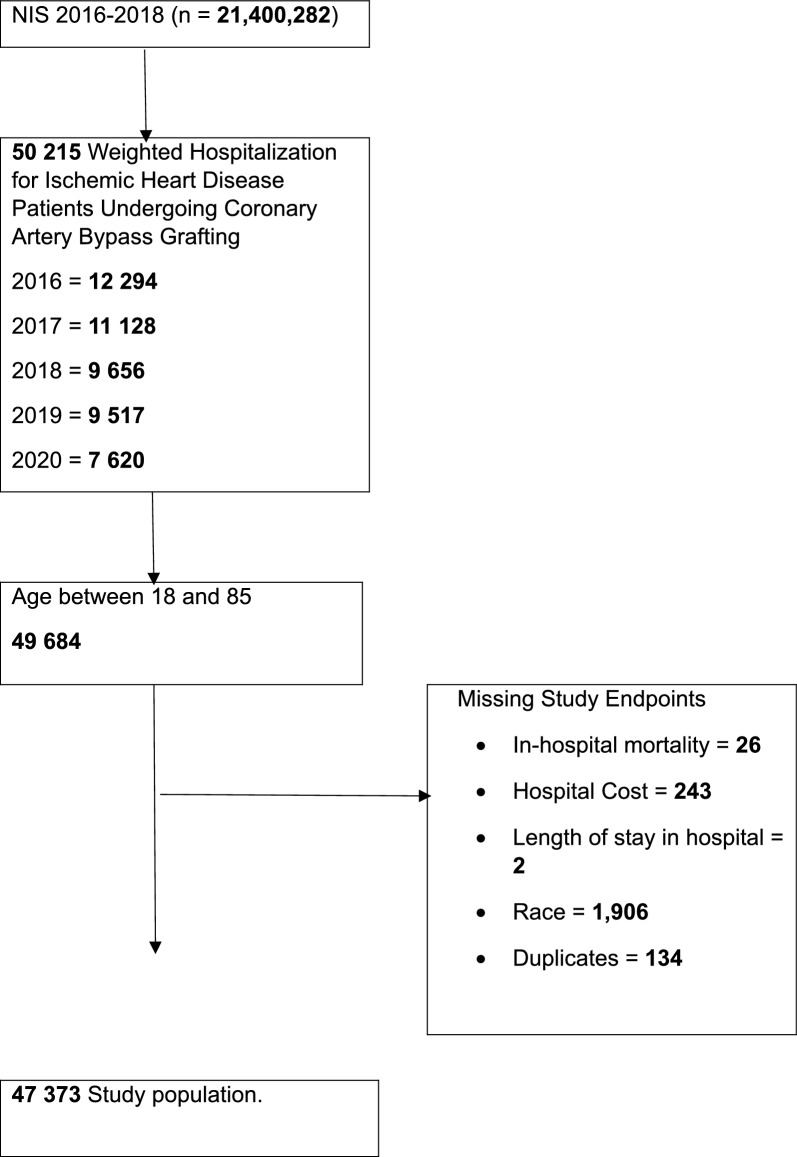
Flow diagram illustrating the selection of the study population from the Nationwide Inpatient Sample (NIS) between 2016 and 2020. Of 50,215 weighted hospitalizations for ischemic heart disease patients undergoing CABG, exclusions were made for missing demographic or outcome data, resulting in a final analytic cohort of 47,373 patients aged 18–85 years

As shown in Fig. 1, minority groups—particularly Black, Hispanic, and Asian/Pacific Islander patients—demonstrated consistently elevated odds of complications, prolonged hospitalization, and higher associated costs when compared with White patients.

### In-hospital mortality

Compared with White patients, Black patients had a modestly elevated but not statistically significant odds of in-hospital mortality (aOR 1.25, 95% CI: 0.96–1.59, p = 0.082). Similarly, Hispanic (aOR 1.23, 95% CI: 0.95–1.55, p = 0.093), Asian or Pacific Islander (aOR 1.20, 95% CI: 0.80–1.73, p = 0.344), and Other patients (aOR 1.23, 95% CI: 0.80–1.80, p = 0.312) did not differ significantly from Whites, whereas Native American patients demonstrated a trend toward higher mortality (aOR 1.86, 95% CI: 0.91–3.34, p = 0.056).

### Unfavourable discharge

Racial disparities were pronounced in unfavourable discharge outcomes. Black patients had 37% higher odds of unfavourable discharge compared with Whites (aOR 1.37, 95% CI: 1.27–1.48, p < 0.001). In contrast, Hispanic patients had significantly lower odds (aOR 0.91, 95% CI: 0.85–0.97, p = 0.0006). Asian or Pacific Islander patients showed no significant difference (aOR 1.07, 95% CI: 0.96–1.20, p = 0.204), while Native American patients had substantially lower odds (aOR 0.47, 95% CI: 0.36–0.60, p < 0.001). The Others group did not differ significantly (aOR 0.92, 95% CI: 0.82–1.04, p = 0.180).

### Prolonged length of stay (LOS)

Prolonged LOS (defined as exceeding the 75th percentile) varied significantly by race (p < 0.0001). Black, Hispanic, and Asian or Pacific Islander patients had 54%, 23%, and 26% higher odds, respectively, of prolonged LOS (aORs: 1.54, 1.23, and 1.26; all p < 0.001). In contrast, Native American patients showed no significant difference (aOR 0.96, 95% CI: 0.72–1.29, p = 0.822), while the others group had 32% higher odds of prolonged LOS (aOR 1.32, 95% CI: 1.16–1.51, p < 0.001).

### Complications

The likelihood of postoperative complications also differed by race (p < 0.001). Black patients had 35% higher odds of complications compared with Whites (aOR 1.35, 95% CI: 1.25–1.46, p < 0.001), Hispanic patients 19% higher (aOR 1.19, 95% CI: 1.10–1.28, p < 0.001), and Asian or Pacific Islander patients 15% higher (aOR 1.15, 95% CI: 1.03–1.29, p = 0.012). In contrast, Native American patients did not differ significantly (aOR 1.02, 95% CI: 0.78–1.31, p = 0.879), and the others group had 18% higher odds of complications (aOR 1.18, 95% CI: 1.05–1.33, p = 0.006).

### Hospital cost

Linear regression analysis of hospital costs (expressed per $1,000) revealed significant disparities (p < 0.0001). Relative to White patients, hospital costs were significantly higher for Black patients (an increase of $17,392.3, p < 0.001), Hispanic patients ($73,447.4, p < 0.001), Asian or Pacific Islander patients ($72,074.5, p < 0.001), and patients categorized as Others ($52,055.8, p < 0.001). In contrast, the cost difference for Native American patients was not statistically significant ($312.3, p = 0.974).

## Discussion

This study highlights significant racial and ethnic disparities in the demographic characteristics, clinical profiles, and postoperative outcomes of IHD patients undergoing CABG in the United States. These patterns are illustrated in Fig. 1, which provides a visual summary of the differential risks and cost burdens experienced by minority groups. Despite adjustments for socioeconomic status, insurance coverage, comorbidity burden, and hospital characteristics, minority groups particularly Black, Hispanic, Native American, and Asian/Pacific Islander patients consistently experienced worse surgical outcomes compared to White patients. These findings reflect the complex interplay of structural, clinical, and socioeconomic factors driving inequity in cardiovascular surgical care.

The predominance of patients aged ≥ 65 years is consistent with the well-established association between aging and increased IHD incidence due to progressive atherosclerosis, endothelial dysfunction, and the cumulative burden of risk factors such as hypertension, diabetes, and dyslipidemia [19, 20]. Conversely, adults aged 18–44 accounted for only a small proportion of CABG cases, reflecting the lower prevalence of advanced multivessel coronary disease in younger individuals and the greater likelihood of these patients receiving percutaneous coronary intervention (PCI) rather than surgical revascularization [21, 22]. However, the relatively higher proportion of younger minority patients undergoing CABG suggests an earlier onset of IHD in these populations, likely driven by disproportionate comorbidity burdens, socioeconomic disadvantages, and limited access to preventive cardiovascular care [23, 24].

Insurance status differed markedly across racial groups. While Medicare and Medicaid were the predominant payers in the overall cohort, private insurance was more common among White and Asian/Pacific Islander patients. In contrast, Black, Hispanic, and Native American patients were more likely to rely on public insurance programs, which are often associated with restricted access to high-quality preoperative care and surgical centres [25]. Previous research has shown that insurance type influences not only access to CABG but also postoperative morbidity, hospital LOS, and long-term survival [26–28]. Even after adjusting for hospital factors and comorbidities, disparities persist, suggesting the contribution of delayed referrals, disease severity at presentation, and potential implicit bias in clinical decision-making [29, 30].

Socioeconomic status, as indicated by household income quartiles, revealed parallel disparities. Patients from the lowest income quartiles were disproportionately represented among Black and Hispanic populations, whereas White and Asian/Pacific Islander patients were more often in the higher income brackets. These findings align with national data showing historical and systemic barriers to wealth accumulation and healthcare access in minority communities [31]. Lower income is associated with delayed treatment, higher comorbidity burden, and reduced access to postoperative rehabilitation all of which negatively influence surgical outcomes [32–34]. Furthermore, hospitals serving low-income and minority communities are often under-resourced, compounding the risk of poor outcomes even after controlling for clinical variables [35, 36].

Differences in admission type were also notable. Elective admissions were more common among White and Asian/Pacific Islander patients, while Black, Hispanic, and Native American patients experienced higher rates of non-elective CABG. This may reflect disparities in outpatient care access, health-seeking behaviour, or delays in diagnosis and referral. Urgent or emergent procedures are associated with worse postoperative outcomes and increased mortality [37–39]. Contributing factors may include reduced primary care access, lower health literacy, and structural barriers such as transportation, insurance coverage, and implicit bias in healthcare delivery [40, 41].

Comorbidity burden also differed by race, with a greater proportion of minority patients particularly Black and Hispanic individuals presenting with a CCI ≥ 3. The heavier comorbidity burden observed in Black and Hispanic patients (Charlson score ≥ 3) likely contributes to their higher complication rates and prolonged LOS, consistent with prior CABG disparity literature [42–44] demonstrating higher rates of chronic diseases in these populations, including diabetes, hypertension, and chronic kidney disease, which complicate surgical management and recovery [45]. These disparities are influenced by social determinants of health, healthcare access limitations, and structural racism [46]. Higher comorbidity burden is associated with greater perioperative risk, increased complications, prolonged hospital stays, and higher readmission rates [47, 48].

Hospital-related differences further reflect structural inequities. White patients were more frequently treated in large, urban teaching hospitals, whereas Black, Hispanic, and Native American patients were more likely to be admitted to smaller or non-teaching facilities. These patterns are consistent with longstanding evidence that minority groups are more likely to receive care at under-resourced hospitals with limited infrastructure and specialized services [49, 50]. In addition, the higher frequency of weekend admissions among minority patients raises concerns about the"weekend effect,"whereby patients admitted during off-hours face increased risk of adverse outcomes due to reduced staffing and access to specialized care [51, 52].

In multivariable analyses, Hispanic and Native-American patients were less likely than White patients to be discharged to skilled-nursing or rehabilitation facilities. Prior work suggests that Hispanic patients often rely on stronger family support networks and may prefer home-based convalescence, a phenomenon sometimes termed the ‘Hispanic paradox’ in discharge disposition [53]. For Native-American patients, the scarcity of post-acute-care facilities in reservation and rural areas can necessitate direct discharge home despite similar functional needs, leading to an apparently protective odds ratio [54, 55].

These systemic inequities translated into significant differences in surgical outcomes. While in-hospital mortality was generally low, Native American patients exhibited the highest rate (3.3%), followed by Black and Hispanic groups. Although these differences did not reach statistical significance after adjustment, the trend is clinically relevant and consistent with earlier studies reporting elevated mortality in minority CABG patients [56, 57].

Secondary outcomes revealed more pronounced disparities. Black patients had significantly higher adjusted odds of unfavorable discharge disposition (aOR 1.37), prolonged LOS (aOR 1.54), and postoperative complications (aOR 1.35) compared to White patients. Hispanic and Asian/Pacific Islander patients also experienced elevated risks of prolonged hospitalization and postoperative morbidity. These trends may reflect not only clinical differences, but also systemic barriers such as language discordance, lower health literacy, and lack of culturally sensitive care [58, 59].

Hospitalization costs were significantly higher among minority patients particularly Hispanic and Asian/Pacific Islander groups despite the overall lower resource availability in facilities serving these populations. These elevated costs likely stem from longer hospital stays, more frequent complications, and less coordinated perioperative care [60].

It is important to interpret the reported odds ratios with caution, particularly for outcomes with high prevalence (e.g., postoperative complications and unfavorable discharge), as odds ratios may overestimate the true relative risk in such cases. Although logistic regression was appropriate for our binary endpoints and adjustment needs, we acknowledge this limitation and recommend that future studies consider using alternative methods such as log-binomial regression or marginal effect estimation to better quantify absolute risk differences.

Observed racial and socioeconomic disparities in postoperative outcomes appear multifactorial. Structural factors such as lower hospital CABG volumes, limited access to high-intensity peri-operative care, and delayed presentation intersect with patient-level barriers (comorbidity burden, reduced social support, and lower access to cardiac rehabilitation) [61, 62] Implicit bias and differential referral patterns may further exacerbate non-elective admissions among minority groups, compounding risk. These mechanisms likely underpin the higher odds of complications, prolonged LOS, and non-home discharge seen in Black and Hispanic patients in our cohort. Collectively, our findings illustrate the intersection of race, socioeconomic status, comorbidity burden, and healthcare system structure in shaping outcomes for patients undergoing CABG. Addressing these disparities will require a multifaceted approach, including improved access to preventive care, expansion of equitable healthcare infrastructure, culturally competent care delivery, and institutional efforts to dismantle structural racism and reduce implicit bias in surgical care pathways.

## Limitations

This study relies on administrative data, which are prone to coding errors and lack key clinical details (ejection fraction, coronary anatomy, intra-operative metrics such as graft number, pump strategy, and cross-clamp time). Race/ethnicity were self-reported or administratively assigned, introducing possible misclassification, and the cross-sectional design precludes assessment of long-term outcomes. Because operative risk scores (e.g., STS) are absent in the NIS, comorbidity was approximated with the Charlson Index, an approach that may under-represent CABG-specific risk. Also, several specific complications were infrequent in some racial strata, complications were analysed as a composite endpoint, which may mask heterogeneity among individual events.

Despite the adjustment for a broad set of demographics, socioeconomic, clinical, and hospital covariates, important drivers of outcome, hospital CABG volume, surgeon experience, peri-operative protocol adherence, medication compliance, functional status, social support, and access to cardiac rehabilitation, were unavailable. Their omission may bias effect estimates and either attenuate or amplify observed disparities. It’s also important to recognize that our analysis did not incorporate innate biological traits or hereditary influences—factors that, while unchangeable, may still shape perioperative risk and clinical trajectories. For instance, variations in inflammatory response pathways or inherited susceptibility to atherosclerosis might partly explain outcome differences across racial groups. Because the NIS dataset does not capture such detailed physiological metrics, we were unable to explore these dimensions directly. Even so, acknowledging their potential role adds a layer of interpretive depth when considering the drivers behind observed disparities.

Finally, some covariates in our models (insurance status, income, hospital type) may function as mediators rather than pure confounders, raising the possibility of over-adjustment and underestimation of disparity magnitude. Future work linking administrative records to clinical registries or prospective cohorts and employing formal mediation analysis, will be essential to address these residual sources of bias.

## Conclusion

This study highlights persistent and multifaceted racial and ethnic disparities in the surgical care and outcomes of IHD patients undergoing CABG in the United States. Minority populations—particularly Black, Hispanic, Native American, and Asian/Pacific Islander patients—faced higher comorbidity burdens, lower access to elective procedures, treatment at less-resourced hospitals, and poorer postoperative outcomes, even after adjustment for socioeconomic and clinical factors. These findings underscore the need for systemic reforms to ensure equitable access to high-quality cardiovascular care. Interventions should focus on expanding preventive services, improving care coordination in underserved communities, addressing social determinants of health, and implementing structural changes to eliminate implicit bias and institutional inequities within the healthcare system.

## Supplementary Information

Additional file 1.

Additional file 2.

## Data Availability

The data utilized in this study are available on the website https://hcup-us.ahrq.gov/nisoverview.jsp which can be made available upon reasonable request.

## Abbreviations

IHD: Ischemic heart disease
CAD: Coronary artery disease
CHD: Coronary heart disease
PCI: Percutaneous coronary intervention
CABG: Coronary artery bypass grafting
NIS: Nationwide inpatient sample
ICD-10: International classification of diseases, 10th revision
CCI: Charlson comorbidity index
CI: Confidence interval
aOR: Adjusted odds ratio
LOS: Length of stay
ANOVA: Analysis of variance
IQR: Interquartile range
