# Supplementary figures and images for: Racial and socioeconomic disparities in postoperative outcomes following coronary artery bypass grafting: a national inpatient analysis
Source: Egypt Heart J. 2025 Aug 12;77:80. doi: 10.1186/s43044-025-00675-7 (PMC12344070; doi:10.1186/s43044-025-00675-7)

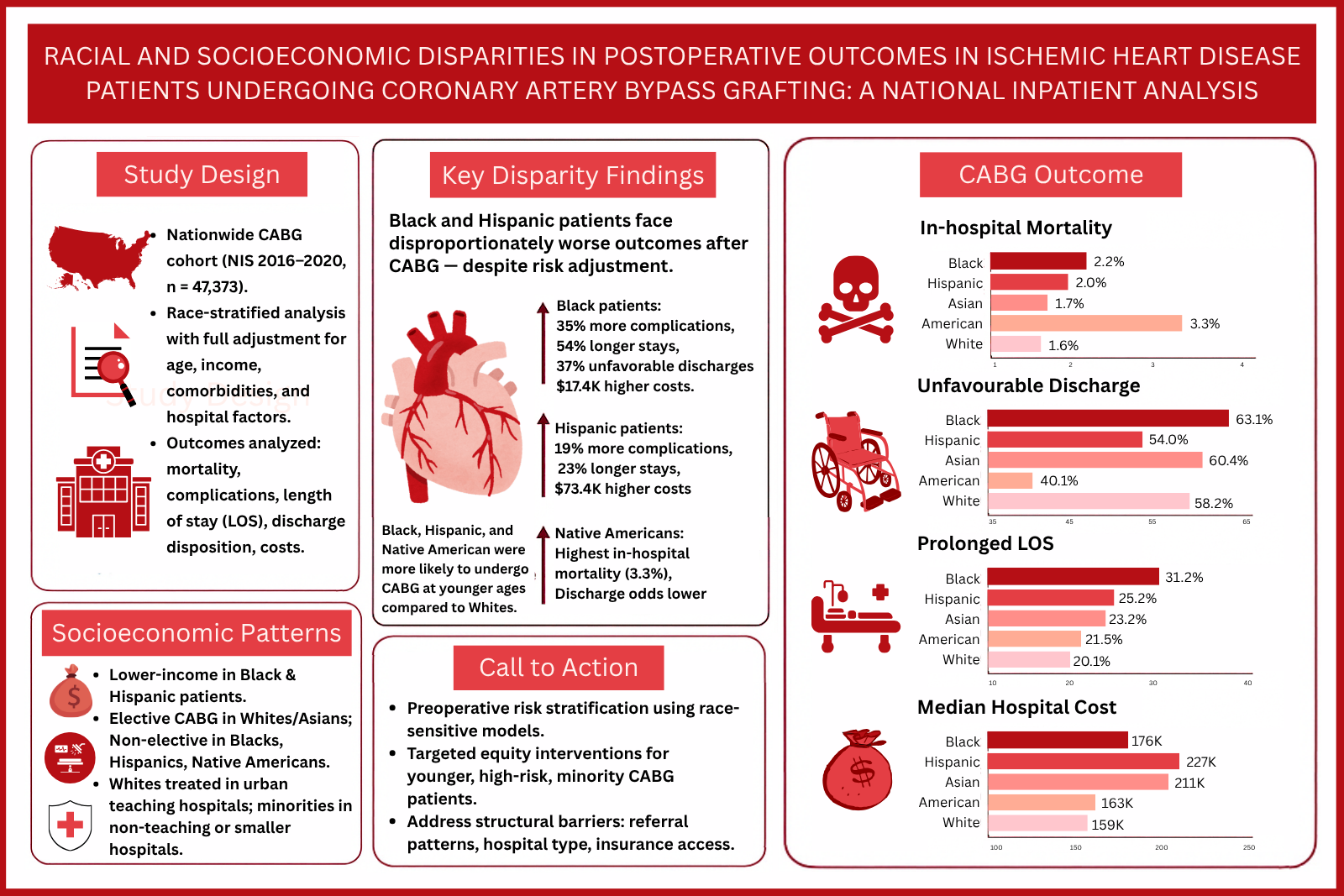

Supplement: Supplementary file 1 — Additional file 1. [file 43044_2025_675_MOESM1_ESM.png]
