# Supplementary material for: Racial and socioeconomic disparities in postoperative outcomes following coronary artery bypass grafting: a national inpatient analysis
Source: Egypt Heart J. 2025 Aug 12;77:80. doi: 10.1186/s43044-025-00675-7 (PMC12344070; doi:10.1186/s43044-025-00675-7)
Supplement: Supplementary file 2 — Additional file 2. [file 43044_2025_675_MOESM2_ESM.docx]

| NIS 2016-2018 (n = **21,400,282**) |
| --- |

| **50 215** Weighted Hospitalization for Ischemic Heart Disease Patients Undergoing Coronary Artery Bypass Grafting  2016 = **12 294**  2017 = **11 128**  2018 = **9 656**  2019 = **9 517**  2020 = **7 620** |
| --- |

| Age between 18 and 85  **49 684** |
| --- |
| Missing Study Endpoints   - In-hospital mortality = **26** - Hospital Cost = **243** - Length of stay in hospital = **2** - Race = **1,906** - Duplicates = **134** |

| **47 373** Study population. |
| --- |

**Fig. 1: Flow Diagram of Study Selection**

**Supplementary table 1: ICD-10 Procedure Codes for CABG of Adults aged 18-85 years with a primary diagnosis of IHD**

| **Coronary Artery Bypass Grafting Group** | **Procedure Codes** |
| --- | --- |
| **One artery** | 02104JW, 02104K9, 02104Z3, 02104Z8, 02104Z9, 02104ZC |
| **Two arteries** | 0211083, 0211089, 021108W, 0211093, 0211098, 0211099, 021109C, 021109F, 021109W, 02110A3, 02110A8, 02110A9, 02110AC, 02110AW, 02110J8, 02110JW, 02110K3, 02110K9, 02110KC, 02110KW, 02110Z3, 02110Z8, 02110Z9, 02110ZC, 0211344, 02113D4, 0211489, 0211493, 0211499, 021149W, 02114A3, 02114A8, 02114A9, 02114JC, 02114KW, 02114Z3, 02114Z9 |
| **Three arteries** | 021208W, 0212093, 0212098, 0212099, 021209C, 021209F, 021209W, 02120A3, 02120A8, 02120A9, 02120AC, 02120AW, 02120J3, 02120J9, 02120JW, 02120K3, 02120KW, 02120Z3, 02120Z8, 02120Z9, 02120ZC, 02120ZF, 0212493, 0212499, 021249W, 02124A3, 02124D4, 02124Z3, 02124Z9 |
| **Four or more arteries** | 0213093, 0213098, 0213099, 021309C, 021309F, 021309W, 02130A3, 02130A8, 02130A9, 02130AC, 02130AW, 02130K3, 02130K8, 02130K9, 02130KW, 02130Z3, 02130Z8, 02130Z9, 02130ZC, 0213493, 0213499, 021349C, 021349W, 02134A9, 02134AW, 02134Z9 |

**Supplementary Table 2:** The International Classification of Diseases, Tenth Revision, Clinical Modification (ICD-10-CM) and International Classification of Diseases, 10th Revision, Procedure Coding System (ICD-10-PCS) codes for In-hospital complications.

| **Perioperative outcomes** | **ICD-10-CM and ICD-10-PCS** |
| --- | --- |
| **Major adverse cardiovascular event (MACE)** |  |
| Myocardial infarction (MI) | I97.710, I97.711, I97.120, I97.121, I46.9 |
| Stroke | I60.9, I61.9, I63.22, I63.139, I63.239, I63.019, I63.119, I63.219, [I97.811](https://www.icd10data.com/ICD10CM/Codes/I00-I99/I95-I99/I97-/I97.811), I97.820, [I97.821](https://www.icd10data.com/ICD10CM/Codes/I00-I99/I95-I99/I97-/I97.821), I97.810 |
| Postprocedural cardiogenic shock | T81.11XA |
| Postprocedural heart failure | I97.130, I97.131 |
| Postprocedural cardiac insufficiency | I97.110, I97.111 |
| **MI** | I97.710, I97.711, I97.120, I97.121, I46.9 |
| **Stroke** | I60.9, I61.9, I63.22, I63.139, I63.239, I63.019, I63.119, I63.219, [I97.811](https://www.icd10data.com/ICD10CM/Codes/I00-I99/I95-I99/I97-/I97.811), I97.820, [I97.821](https://www.icd10data.com/ICD10CM/Codes/I00-I99/I95-I99/I97-/I97.821), I97.810 |
| **Transient ischemic attack (TIA)** | G45.9, I67.848 |
| **Neurological complications** |  |
| Nervous system complication, unspecified | [G97.81](https://www.icd10data.com/ICD10CM/Codes/G00-G99/G89-G99/G97-/G97.81) |
| Central nervous system complication | G97.81, G97.82 |
| Iatrogenic cerebrovascular infarction or hemorrhage | [I97.811](https://www.icd10data.com/ICD10CM/Codes/I00-I99/I95-I99/I97-/I97.811), [I97.821](https://www.icd10data.com/ICD10CM/Codes/I00-I99/I95-I99/I97-/I97.821),  I97.810, I97.820 |
| Transient ischemic attack | [G45.9](https://www.icd10data.com/ICD10CM/Codes/G00-G99/G40-G47/G45-/G45.9), [I67.848](https://www.icd10data.com/ICD10CM/Codes/I00-I99/I60-I69/I67-/I67.848) |
| Any stroke | 160.9, 161.9, 163.22, 163.139, 163.239  163.019, 163.119, 163.219 |
| **Pericardial complications** |  |
| Hemopericardium | I31.2 |
| Tamponade | [I31.4](https://www.icd10data.com/ICD10CM/Codes/I00-I99/I30-I52/I31-/I31.4) |
| Pericardiocentesis | 0W9DXXX, 0W9CXXX, 0W9D40Z |
| Acute pericarditis | I30.1, I30.8, I30.9 |
| **Pacemaker implantation** | 0JH606Z, 0JH636Z, 0JH806Z, 0JH836Z, 0JH60PZ, 0JH63PZ, 0JH80PZ, 0JH83PZ, 0JH604Z, 0JH634Z, 0JH804Z, 0JH834Z, 0JH605Z, 0JH635Z, 0JH805Z, 0JH835Z, 02H73KZ, 02HK3KZ, 02HL3KZ, 02HN0KZ, 02HN4KZ, 0JH608Z, 0JH638Z, 0JH808Z, 0JH838Z, 02H60KZ, 02H63KZ, 02H64KZ, 02H70KZ, 02H73KZ, 02H74KZ, 02HK0KZ, 02HK3KZ, 02HK4KZ, 02HL0KZ, 02HL3KZ, 02HL4KZ, 0JH608Z, 0JH638Z, 0JH808Z, 0JH838Z, 02H60KZ, 02H63KZ, 02H64KZ, 02H70KZ, 02H73KZ, 02H74KZ, 02HK0KZ, 02HK3KZ, 02HK4KZ, 02HL0KZ, 02HL3KZ, 02HL4KZ, 0JH608Z, 0JH638Z, 0JH808Z, 0JH838Z, |
| **Cardiogenic shock** | R57.0 |
| **Respiratory complications** |  |
| Pneumothorax/hemothorax | [J95.811](https://www.icd10data.com/ICD10CM/Codes/J00-J99/J95-J95/J95-/J95.811), [J95.812](https://www.icd10data.com/ICD10CM/Codes/J00-J99/J95-J95/J95-/J95.812), J95.830, J95.831, J94.2 |
| Diaphragm paralysis | [J98.6](https://www.icd10data.com/ICD10CM/Codes/J00-J99/J96-J99/J98-/J98.6) |
| Post-operative Respiratory Failure | [J95.821](https://www.icd10data.com/ICD10CM/Codes/J00-J99/J95-J95/J95-/J95.821), [J96.00](https://www.icd10data.com/ICD10CM/Codes/J00-J99/J96-J99/J96-/J96.00), [J95.822](https://www.icd10data.com/ICD10CM/Codes/J00-J99/J95-J95/J95-/J95.822), [J96.20](https://www.icd10data.com/ICD10CM/Codes/J00-J99/J96-J99/J96-/J96.20) |
| Pulmonary insufficiency | J95.2, J95.3 |
| Respiratory arrest | R09.2 |
| Other iatrogenic Respiratory Complications | J95.88, J95.89, J95.850, J95.851, J95.859 |
| **Mechanical ventilation** | 5A1935Z, 5A1945Z, 5A1955Z |
| **Acute kidney injury (AKI)** | N17.9, N17.0, N17.1, N17.2 |
| **Post-procedural renal failure** | N99.0 |
| **Venous thromboembolism (VTE)** | I82.401, I82.402, I82.403, I82.409, I82.411, I82.412, I82.413, I82.419, I82.421, I82.422, I82.423, I82.429, I82.431, I82.432, I82.433, I82.439, I82.441, I82.442, I82.443, I82.449, I82.451, I82.452, I82.453, I82.459, I82.461, I82.462, I82.463, I82.469, I82.491, I82.492, I82.493, I82.499, I82.4Y1, I82.4Y2, I82.4Y3, I82.4Y9, I82.4Z1, I82.4Z2, I82.4Z3, I82.4Z9 |
| **Pulmonary embolism (PE)** | I26.02, I26.09, I26.92, I26.93, I26.94 |
| **Hemorrhage/hematoma** |  |
| Hemorrhage/hematoma complicating a procedure | [I97.411](https://www.icd10data.com/ICD10CM/Codes/I00-I99/I95-I99/I97-/I97.411), [I97.418](https://www.icd10data.com/ICD10CM/Codes/I00-I99/I95-I99/I97-/I97.418), [I97.42](https://www.icd10data.com/ICD10CM/Codes/I00-I99/I95-I99/I97-/I97.42), [I97.611](https://www.icd10data.com/ICD10CM/Codes/I00-I99/I95-I99/I97-/I97.611)  [I97.618](https://www.icd10data.com/ICD10CM/Codes/I00-I99/I95-I99/I97-/I97.618), [I97.620](https://www.icd10data.com/ICD10CM/Codes/I00-I99/I95-I99/I97-/I97.620), [I97.411](https://www.icd10data.com/ICD10CM/Codes/I00-I99/I95-I99/I97-/I97.411), [I97.418](https://www.icd10data.com/ICD10CM/Codes/I00-I99/I95-I99/I97-/I97.418), [I97.42](https://www.icd10data.com/ICD10CM/Codes/I00-I99/I95-I99/I97-/I97.42), [I97.621](https://www.icd10data.com/ICD10CM/Codes/I00-I99/I95-I99/I97-/I97.621), [I97.631](https://www.icd10data.com/ICD10CM/Codes/I00-I99/I95-I99/I97-/I97.631), [I97.638](https://www.icd10data.com/ICD10CM/Codes/I00-I99/I95-I99/I97-/I97.638) |
| Acute post-hemorrhagic anemia | D62 |
| Hemorrhage requiring transfusion | 3023XXX, 3024XXX |
| **Infection** |  |
| Fever | T82.6, T82.7, R50.82 |
| Septicemia | A41.9, A65.20, [T81.12XA](https://icd.codes/icd10cm/T8112XA) |
| Post-procedural aspiration pneumonia | J95.89 |
| **Sepsis** | T81.12XA, T81.44XA |
| **Deep wound complication** | T81.32XA, T81.43XA |
| **Superficial wound complications** | L76.34, L76.32, T81.31XA, T81.41XA, T81.42XA, T81.40XA |
| **Vascular complication** |  |
| Accidental puncture or laceration during a procedure | [I97.51](https://www.icd10data.com/ICD10CM/Codes/I00-I99/I95-I99/I97-/I97.51), [I97.52](https://www.icd10data.com/ICD10CM/Codes/I00-I99/I95-I99/I97-/I97.52) |
| Injury to blood vessels | S25.X, S35.X |
| Arteriovenous Fistula | I77.0 |
| Injury to retroperitoneum | [S36.899A](https://www.icd10data.com/ICD10CM/Codes/S00-T88/S30-S39/S36-/S36.899A) |
| Vascular complication requiring surgical/percutaneous repair | [03QY0ZZ](https://www.icd10data.com/ICD10PCS/Codes/0/3/Q/Y/03QY0ZZ), [03QY3ZZ](https://www.icd10data.com/ICD10PCS/Codes/0/3/Q/Y/03QY3ZZ), [03QY4ZZ](https://www.icd10data.com/ICD10PCS/Codes/0/3/Q/Y/03QY4ZZ), [04QY0ZZ](https://www.icd10data.com/ICD10PCS/Codes/0/4/Q/Y/04QY0ZZ), [04QY3ZZ](https://www.icd10data.com/ICD10PCS/Codes/0/4/Q/Y/04QY3ZZ), [04QY4ZZ](https://www.icd10data.com/ICD10PCS/Codes/0/4/Q/Y/04QY4ZZ), [05QY0ZZ](https://www.icd10data.com/ICD10PCS/Codes/0/5/Q/Y/05QY0ZZ), [05QY3ZZ](https://www.icd10data.com/ICD10PCS/Codes/0/5/Q/Y/05QY3ZZ), [05QY4ZZ](https://www.icd10data.com/ICD10PCS/Codes/0/5/Q/Y/05QY4ZZ), [06QY0ZZ](https://www.icd10data.com/ICD10PCS/Codes/0/6/Q/Y/06QY0ZZ), [06QY3ZZ](https://www.icd10data.com/ICD10PCS/Codes/0/6/Q/Y/06QY3ZZ), [06QY4ZZ](https://www.icd10data.com/ICD10PCS/Codes/0/6/Q/Y/06QY4ZZ), [02QW0ZZ](https://www.icd10data.com/ICD10PCS/Codes/0/2/Q/W/02QW0ZZ), [02QW3ZZ](https://www.icd10data.com/ICD10PCS/Codes/0/2/Q/W/02QW3ZZ), [02QX4ZZ](https://www.icd10data.com/ICD10PCS/Codes/0/2/Q/X/02QX4ZZ), [03Q00ZZ](https://www.icd10data.com/ICD10PCS/Codes/0/3/Q/0/03Q00ZZ), [03Q03ZZ](https://www.icd10data.com/ICD10PCS/Codes/0/3/Q/0/03Q03ZZ), 03Q04ZZ, 03Q10ZZ, 03Q13ZZ, 03Q14ZZ, 03Q20ZZ, 03Q23ZZ, 03Q24ZZ, 03Q30ZZ, 03Q40ZZ, 03Q33ZZ, 03Q43ZZ, 03Q44ZZ, 03Q50ZZ, 03Q53ZZ, 03Q54ZZ, 03Q60ZZ, 03Q63ZZ, 03Q64ZZ, 03Q74ZZ 03Q70ZZ, 03Q73ZZ, 03Q80ZZ, 03Q83ZZ, 03Q84ZZ, 03Q90ZZ, 03Q93ZZ, 03Q94ZZ, 03QA0ZZ, 03QA3ZZ, 03QA4ZZ, 03QB0ZZ, 03QB3ZZ, 03QB4ZZ,03QC0ZZ, 03QC3ZZ, 03QC4ZZ,03QY0ZZ, 03QY3ZZ,03QY4ZZ,04Q00ZZ,  04Q03ZZ, 04QC0ZZ, 04QC3ZZ, 04Q04ZZ, 04QC4ZZ,04QD0ZZ, 04QD3ZZ,04QD4ZZ, 04QE0ZZ 04QE3ZZ, 04QE4ZZ, 04QF0ZZ, 04QF3ZZ, 04QF4ZZ, 04QH0ZZ, 04QH3ZZ, 04QH4ZZ,04QJ0ZZ, 04QJ3ZZ, 04QJ4ZZ,04QK0ZZ, 04QK3ZZ, 04QL0ZZ, 04QL3ZZ, 04QL4ZZ, 04QY0ZZ, 04QY3ZZ |
| Hemorrhage from vascular procedures | T82.838, T82.837 |
| Other artery and vein complications | T81.72XA, T81.719A |
| **Diaphragmatic paralysis** | J98.6 |
| **Reopen surgery** | 0W390ZZ, 0W3B0ZZ, 0W3C0ZZ, 0W3D0ZZ, 0W3Q0ZZ |
